# NeuronAlg: An Innovative Neuronal Computational Model for Immunofluorescence Image Segmentation

**DOI:** 10.3390/s23104598

**Published:** 2023-05-09

**Authors:** Giuseppe Giacopelli, Michele Migliore, Domenico Tegolo

**Affiliations:** 1 National Research Council, Institute of Biophysics, 90153 Palermo, Italy; michele.migliore@cnr.it; 2 Dipartimento Matematica e Informatica, Universitá degli Studi di Palermo, 90123 Palermo, Italy

**Keywords:** biomedical imaging, explainable ai, neuron physiology networks, computer-aided analysis, image segmentation, pattern analysis

## Abstract

*Background*: Image analysis applications in digital pathology include various methods for segmenting regions of interest. Their identification is one of the most complex steps and therefore of great interest for the study of robust methods that do not necessarily rely on a machine learning (ML) approach. *Method*: A fully automatic and optimized segmentation process for different datasets is a prerequisite for classifying and diagnosing indirect immunofluorescence (IIF) raw data. This study describes a deterministic computational neuroscience approach for identifying cells and nuclei. It is very different from the conventional neural network approaches but has an equivalent quantitative and qualitative performance, and it is also robust against adversative noise. The method is robust, based on formally correct functions, and does not suffer from having to be tuned on specific data sets. *Results*: This work demonstrates the robustness of the method against variability of parameters, such as image size, mode, and signal-to-noise ratio. We validated the method on three datasets (Neuroblastoma, NucleusSegData, and ISBI 2009 Dataset) using images annotated by independent medical doctors. *Conclusions*: The definition of deterministic and formally correct methods, from a functional and structural point of view, guarantees the achievement of optimized and functionally correct results. The excellent performance of our deterministic method (NeuronalAlg) in segmenting cells and nuclei from fluorescence images was measured with quantitative indicators and compared with those achieved by three published ML approaches.

## 1. Introduction

One of the objectives of automatic image analysis is the formalization of methodologies to identify quantitative indicators that characterize elements on pathological slides. The analysis of immunofluorescence images is becoming a fundamental tool for identifying predictive and prognostic elements that can be used to diagnose various pathologies. Cell studies, particularly cancer cell studies, could allow the identification of predictive parameters, to improve patient diagnosis and develop prognostic tests. The segmentation phase for single cells and nuclei is relatively simple if performed by an expert on cytological images, because, in most cases, cells/nuclei are intrinsically separated from each other. Automated recognition combined with unsupervised and automatic quantitative analysis helps doctors in decision-making and provides cognitive support during the diagnosis of pathologies carried out on slides [1]. This reduces the level of subjectivity, which may affect the decision-making process. The segmentation process is a necessary first step in obtaining quantitative results from cellular or nuclear images. An accurate and detailed segmentation, in which single instances of cells/nuclei are highlighted, would provide a valuable starting point for identifying their quantitative characteristics. Incorrect biomedical conclusions may result from the inability of algorithms to separate different and more complex aggregations of cells/nuclei [2]. For example, complex images, such as those shown in Figure 1, can result in incorrect conclusions about specific instances or, in some cases, in disregarding fundamental aggregates that would allow obtaining a more accurate diagnosis. Precise segmentation allows highlighting the instances on which to focus attention, thus improving the diagnostic process. This process is not the only motivation for the development of new methodologies for extracting characteristics from digital pathological images, and a better understanding of the pathological processes caused by cellular anomalies would be helpful in clinical and research settings.

In biomedical data analysis, technologies based on big data and machine learning contribute to the identification or prediction of a disease and can improve diagnostic processes and guide doctors towards personalized decision-making [3]. The use of machine learning technologies on biomedical images can hopefully improve clinical processes (diagnostic and prognostic) by reducing human errors. However, in recent years, the use of these methods has highlighted the presence of biases, attributable to distortions in the learning dataset or related algorithms, that lead to an inaccurate decision-making process. For example, a ML algorithm will only be valid for the dataset on which it has been trained; therefore, if there are distortions, these will be reiterated and probably exacerbated by an ML application [4]. Different studies have offered various solutions from different perspectives. Some studies focused on an automated approach to analysis of medical images with consolidated tools [5,6,7,8,9,10,11]. This has proven to be quite useful in clinical cases, such as those based on wavelet transforms, which can extract a set of features and discriminate objects [12,13]. Others have developed innovative methodologies that aim to understand the functional and deterministic correctness of the adopted functions. In De Santis et al. [14] adopted a segmentation strategy based on a grey-level top-hat filter with disk-shaped structuring elements, and a threshold at the 95th percentile. Then, cells are segmented using the ISODATA threshold. This is important because the availability of formally correct methods, from both a functional and structural point of view, guarantees that a method can achieve optimized and functionally correct results.

This study introduces a deterministic method for cell or nuclei segmentation that does not use ML techniques or artificial neural network models and is immune to adversative noise. Our results demonstrate a high degree of robustness, reliability, and computational performance, particularly for noisy images. We believe that this method will significantly improve the ability to understand all phases of the process of identifying regions of interest in immunofluorescence images.

## 2. Related Works

The design and development of intuitive and efficient methodologies for highlighting biological patterns is the mission of a subset of the scientific community. A collaborative framework for managing intelligence modules and complete datasets has grown in the last decade. Wählby et al. [15] presented a contribution on image analysis algorithms for the segmentation of cells imaged using fluorescence microscopy. The method includes an image preprocessing step, a module to detect objects and their merging, and a threshold for the statistical analysis of certain shapes describing the features of the previous results, which allows the splitting of the objects. The authors declared that the method is fully automatic after the training phase on a representative set of training images. It demonstrated a correct segmentation of between 89% and 97%. An interesting study by Rizk et al. [16] presented a versatile protocol for the segmentation and quantification of subcellular shapes. This protocol detects, delineates, and quantifies subcellular structures in fluorescence microscopy images. Moreover, the same protocol allows application to a wide range of biomedical images by changing certain parameter values.

Di Palermo et al. highlighted the use of wavelet transforms applied to the main phases of immunofluorescence image analysis. They tried to utilize the versatility of wavelet transform (WT) for various levels of analysis to classify indirect immunofluorescence images (IIF) and to develop a framework capable of performing image enhancement, ROI segmentation, and object classification [12]. They reported the following success rates (in terms of specificity, sensitivity, and accuracy) of the method for different types of cells, classifying them as mitotic and non-mitotic: CE (98.15, 92.6, 97.9); CS (93.83, 71.1, 91.6); and NU (91.53, 88.1, 91.3).

In order to explicitly deal with the intraclass variations and similarities present in most of HEp-2 cell images datasets, Vununu et al. in [17] proposed a dynamic learning method that uses two deep residual networks with the same structure. First, the results of discrete wavelet transform are archived from the input images. Then, the approximation coefficients are used as inputs for the first network, and a second network has the sum of all coefficients as input. Finally, the results are fused at the end of the process, to combine the information extracted from the two networks.

Such methods are only a few examples of the multitude of image segmentation algorithms introduced in the literature: k-means clustering [18,19], graph cuts [20,21,22], active contours [23,24], and watershed methods [25,26].

An exhaustive dissertation on emerging image segmentation techniques was presented in [27] by Minaee et al., in which approaches based on deep convolutional neural networks and supervised machine learning methods were introduced to solve the segmentation task as a subfield of the more general classification strategies.

Pan et al. presented an efficient framework in which a simplification of U-Net and W-Net formed an original method for nuclei segmentation; they named it attention-enhanced simplified W-net (ASW-Net). This method is based on a light network and a cascade structure of network connections. This infrastructure enables the efficient extraction of features. Furthermore, the adopted post-processing refined the accuracy of the segmentation results [28]. On low-resolution images (909 images), the assessment reported that 89% of the cells were correctly segmented, whereas on high-resolution images (251 images), 93% of cells were correctly segmented; therefore, the method achieved a 89–97% segmentation accuracy.

Van Valen et al. investigated multiple cell types; they used a deep convolutional neural network as a supervised machine learning method, to achieve robust segmentation of the cytoplasm of mammalian cells, as well as of the cytoplasm of individual bacteria. They demonstrated through a standard index (Jaccard index) that their methodology improved on the accuracy of other methods [29]. Therefore, they asserted that deep convolutional neural networks are an accurate method and are generalizable to a variety of cell types, from bacteria to mammalian cells; they also reported a remarkable degree of success in different areas: bacteria 0.95(J.I.); mammalian nuclei 0.89(J.I.); various mammalian cytoplasm from 0.77 to 0.84(J.I.).

Another remarkable approach is using a deep learning method to perform simultaneous segmentation and classification of the nuclei in histology images. The network HoVer-Net [30] is based on the prediction of horizontal and vertical maps of nuclear distances from their centers of mass, to detach the clustered nuclei. Graham et al. stated that the HoVer-Net network achieved at least the same performance as that reported by several recently published methods on multiple H&E histology datasets [30]. Moreover, they experimented with their methodology on different exhaustively annotated datasets and performed instance segmentation without classification, with a Dice percentage ranging from 0.826 to 0.869.

The Residual Inception Channel Attention-Unet, an Unet-based neural network for nuclei segmentation, was proposed in [31] (RIC-Unet). The authors included the techniques of residual blocks, multi-scale, and channel attention mechanisms in RIC-Unet, to achieve a more precise segmentation of nuclei. The effectiveness of this approach was compared to traditional segmentation methods and neural network techniques, as well as tested on different datasets. They reported three quantitative indices (Dice, F1-score, and Jaccard) ranging from 0.8008 to 0.7844, 0.8278 to 0.8155, and 0.5635 to 0.5462, respectively.

Machine learning has had a transformative impact on image segmentation; thus, the scientific community considers it a powerful tool for analyzing biomedical images. However, the training phase, tuning parameters, and statistical learning conditions are ML, AI, and DL processes. In contrast, our method does not require any fine-tuned parameters nor has a training phase, and it is robust in terms of adversative noise.

The evolution of information technologies and high-performance computing power (HPC) combined with new developments in computational neuroscience have highlighted potential applications in many fields: pattern recognition, hardware design for neurons, data mining, and modeling. The modeling of the interconnection between neurons has allowed defining non-canonical computational models able to satisfy processes in applied sciences. Abhilash et al., in [32], presented a review of the recent developments in the field of computational neuroscience. Their contribution supports researchers from other fields of science and engineering who intend to move into the field of computational neuroscience and neuronal communications. Moreover, this field is less disconnected to standard artificial intelligence approaches and more linked to actual neuronal intelligence [33,34,35,36,37].

## 3. Methods and Data

This section describes some machine learning techniques, as well as the set of datasets used to validate the effectiveness and compare our method. Datasets with cells and nuclei are also described. In addition, a brief description of the Otsu algorithm, wavelet transform, and active contour models used in the preprocessing phase of our methodology is provided in Section 3.2.

### 3.1. Machine Learning Approaches

In recent decades, neuronal networks have proven to be a solution to various computational problems [38,39,40] and have achieved considerable success in the segmentation and classification of the objects present in digital images [41,42]. The basic technique consists of automatic learning from a training set, with a multilevel hierarchical strategy; in some cases, this functionality is invariant with respect to small or large variations in the learning samples, producing relevant results [39,43]. Convolutional neural networks (CNN) are among the best performing neuronal networks, consisting of pairs of convolutional levels coupled to connected levels. The fundamental components of a CNN can be summarized as follows: convolutive filters operating within convolutional levels, in which the goal is to produce a map of features from input images; grouping functions, in which the outputs of the convolutional levels converge and in which maximum values are selected; functions for the assignment of the probabilities, in which the data coming from the grouping functions are permitted, and in which the probabilities of the input data belonging to a specific class are assigned [44]. Moreover, Krotov et al. in [45] introduced a learning rule based on biological activities related to the change of synaptic forces. They suggested local changes dependent only on the activities of pre- and postsynaptic neurons. Synaptic weights are learned using only bottom-up signals, and the algorithm operates without knowing the task present at higher levels of the network. Despite this lack of knowledge of the task, the method finds a set of weights that performs well compared to standard feed-forward networks trained end-to-end with a backpropagation algorithm. However, alternative approaches exist for selecting the maximum probability, such as the convolution with strides approach [46] and capsule neural networks [47].

The following section provides a brief description of some of the best and most popular neural networks (NN) for image segmentation. In addition, this section provides comparisons with our method.

#### 3.1.1. U-Net

U-Net is a deep learning network [48] for image processing. The idea is to scale down the information of the input image using convolution layers and then scale up the information through transposed convolutional layers, to obtain an image with the exact resolution of the original image using the information of the semantic segmentation in each pixel. U-Net is the most popular segmentation algorithm for several reasons:

- The architecture is so simple that it can be applied to many medical imaging segmentation tasks [49].
- Even if the network is deep, it can be trained in a short time, requiring low computational resources (the U-Net used in this study [48] was trained on a GPU RTX 2070 with 8 GB of VRAM in approximately 1 h).
- This network requires few computational resources for prediction, and it has a very fast forwarding time [50].

The main disadvantage of this architecture is that the cell/nuclei segmentation task returns a binary label and cannot separate single cells/nuclei by default. In this work, we used the Keras/Tensorflow implementation available at https://github.com/zhixuhao/unet (accessed on 10 January 2022) inspired by [48] with grayscale images at a resolution of 512×512.

#### 3.1.2. KG Network

A keypoint graph network (from now on KG Network) is a neural network based on the concept of the keypoint graph [51]. The network first applies ResNet34-based feature extraction. Then, the network layers identify some points (called keypoints) that discretize the input image, and the collected keypoints are processed to extract the bounding boxes of the cells/nuclei. Finally, the bounding boxes of the cells/nuclei are taken as the input for the final layers, which extract the cell/nuclei masks. This network (in contrast with the previous U-Net) provides object-by-object segmentation. However, it has a more considerable forward time than U-Net and had a training time of approximately 4 h on the same machine with a GPU RTX 2070 with 8 GB of VRAM. In this study, we used a PyTorch implementation publicly available at the website https://github.com/yijingru/KG_Instance_Segmentation (accessed on 10 January 2022) based on Ref. [51], with grayscale images at a resolution of 256×256.

#### 3.1.3. R-CNN

A mask R-CNN uses a region-based convolutional neural network (R-CNN) [52] to extract the masks of single cells and nuclei. This network has a more significant forward time than the previous networks, and it requires a massive amount of VRAM to be trained. In fact, we trained it on a cloud node with a GPU NVIDIA K80 with 24 GB of VRAM for 2 h and 30 min. For this study, we used a Tensorflow/Keras implementation publicly available at https://github.com/matterport/Mask_RCNN (accessed on 10 January 2022) with grayscale images at a resolution of 256×256.

### 3.2. Deterministic Approaches

Several approaches do not use machine learning for object segmentation. However, most of these are characterized by a single feature: they work for a combination of parameters depending on the dataset (in the worst cases, only on a single image), but this combination must be manually found by trial and error. For this reason, these approaches are described below as having a wide prospects in the current state of the art. Although they were not fully tested in this study, a few of these approaches will be used in our pipeline.

#### Otsu’s Method

The first segmentation approach was Otsu’s method [53]. It is based on the simple idea that if a white object is placed on a dark background, then there must be two peaks in the grayscale pixel value histogram [54]: the first is the most common background color, and the second is the most common object color. Thus, the idea behind Otsu’s method is to find a grayscale value $T_{Otsu}$ defining two classes of pixels as

- The class (class 0) of pixels with a grayscale value smaller than $T_{Otsu}$;
- The class (class 1) of pixels with a grayscale value greater than $T_{Otsu}$.

Otsu’s method iterates the threshold $T_{Otsu}$ from 0 to 255, to determine the value that maximizes the variance. It is a powerful tool because it does not require any tuning by the user. However, it has many disadvantages that render it impractical for real-world images. The most common underlying assumption is that a perfectly bright object is placed on a completely dark background. This rarely occurs in real-world images [55]. If the previous hypothesis holds, the color histogram is a perfect bimodal distribution; however, real-world images are noisy, making Otsu’s segmentation difficult. Another common problem is that when the colors of the object and background are easily distinguishable, the object is very small in comparison to the background [56]. In this case, the peak of the object pixels in the histogram exists but is too small to be relevant in terms of variance. This case recurs in the proposed model and is solved using our neuronal agents. In conclusion, Otsu’s method is very powerful, but when used alone, it can lead to poor performance.

### 3.3. Watershed

One of the limitations of Otsu’s method is that it returns a binary result, where every pixel indicates class 1 or class 0. However, in many applications, it is not sufficient to identify the pixel class, and it is also necessary to discriminate between different objects of the same class inside the image. A classic example of this is the analysis of cellular microscopy images. In this case, the user is not only interested in where cells/nuclei are placed, but they are also interested in distinguishing different cells/nuclei, to count or classify them. Therefore, a watershed [57] was introduced. This transform can be used to distinguish homogeneous objects based on the gradient of the image. Several versions of this algorithm have been proposed [58]. We considered the most common; that is, Meyer’s watershed [59], implemented in the open-source framework OpenCV [60]. This transform starts with a set of markers established by the user (most of the time, this is extracted in an automated manner using mathematical morphology). The algorithm performs a “flooding” of the image, in order to find the optimal “basins” using the following procedure:

- The markers are initialized with the user’s input;
- The neighboring pixels of a marked pixel are inserted into the queue with a priority proportional to the gradient modulus of the image of the inserted pixel;
- The pixel with the highest priority is extracted. If the surrounding marked pixels have the same label, the pixel is marked with this label. All the surrounding pixels that are not yet marked are inserted into the queue;
- Return to step 2, until the queue is empty.

Watersheds are one of the simplest algorithms for splitting purposes, and with good quality images, they perform very well. However, this method has several disadvantages. Often, the initial markers are selected starting from Otsu’s thresholding, which means that these implementations can be affected by the same problems as described in the previous section. Another disadvantage is that in noisy images, a watershed can be affected by oversplitting [58], meaning that there are more clusters than expected. In addition, for very close and merged objects (such as cell/nucleus clusters), mathematical morphology methods can fail to separate single objects. For these reasons, the proposed model has some preprocessing steps in the marker individuation and postprocessing steps for the watershed masks, to obtain optimal results.

#### Active Contour Model

The snake or active counter model was widely used in this study. However, our neuronal agents lead to a better generalization without parameter tuning; thus, they replace the snake classic function. The following section describes some aspects of the general snake, to better understand the entire process. The idea of an active contour module is to build a discrete contour made of keypoints (often called a snake) and aimed at minimizing a line functional. To define this line functional, three energies associated with ACM must be defined [61]. The first is $E_{int}$, which is the internal energy functional defined to keep the ACM as rigid as possible and to avoid excessive ACM shrinking. The second energy is the image energy functional, defined as

$$E_{img}=w_{line}E_{line}+w_{edge}E_{edge}+w_{term}E_{term}$$

where $E_{line}$ from the grayscale values of the image, $E_{edge}$ from its gradient, the energy $E_{term}$ taking into account the normal derivative of the image gradient, and the constants $w_{line},w_{edge},w_{term}$ must first be defined. Therefore, ACM minimizes the functional calculated along the contour. $E_{con}$ is an additional component used to guide the user through the process by adding constraints to the run [61]. The main criticisms of this kind of algorithm include the following: Problems involving the internal functional for complex shapes [62]; Convergence of the algorithm to local minima [63]. The first class of problems is caused by the fact that ACM uses $E_{img}$ to attract the snake towards the edge and $E_{int}$ to avoid the collapse of the snake at a single point, keeping it as smooth as possible. The second type of problem is caused by the process of minimization, which can converge to a local minimum (caused, for example, by a source of noise), leading to poor convergence to the actual contour. These problems imposed the definition of a new contour model implemented by our neuronal agents, which is immune to parameter tuning.

### 3.4. Datasets

Fluorescence (F.) is a technique used to detect specific biomolecules within a tissue or cells/nuclei using specific antibodies that contain fluorescent dyes [64]. There are different F. techniques. A distinction can be made between direct and indirect F. [64]: in direct F., the antibody carrying the fluorophore (the fluorescent substance) binds directly to the biomolecule, whereas in indirect F., the antibody carrying the fluorophore binds to other antibodies or molecules that are directed against the biomolecule of choice, thus binding indirectly to it. Our discussion focuses more on the results of F. than on the methods involved in this procedure. The result of F. is a microscopy image that shows the fluorescence of the area bound by the antibody, in contrast to the darker regions not bound by the antibody. The proposed algorithms work with grayscale images; subsequently, we will assume a preprocessing step that extracts such information from each dataset. This assumption generally holds [65], but it is not true for all datasets. For example, some datasets are RGB, and they contain most of their information in the green channel [66] or even in the blue channel [67]. Therefore, in the proposed analysis, it is implied that the starting point is a grayscale version of every dataset with as much information as possible. Here is a brief description of the datasets involved in the experiments.

#### 3.4.1. Neuroblastoma Dataset

The first dataset was the dataset introduced in [65]. It is composed of four samples of tumor tissue and four samples of the bone marrow of Neuroblastoma patients. The dataset was created with the aid of the Children’s Cancer Research Institute (CCRI) biobank (EK.1853/2016), to establish a benchmark for experiments on automatic cell nuclei segmentation. The dataset consists of 41 training images and 38 test images in jpg format. with a resolution variable of approximately 1200×1000 F. cells/nuclei. The images are already in grayscale, which means that brighter zones are white and darker zones are black, so they do not require preprocessing. Segmentation was performed manually by the authors [65], and this distinguished different cells/nuclei. The segmentation was stored in text-based files. This dataset has proven to be a sufficiently hard benchmark to test models on real-world images [68]. For these reasons, it was considered the main benchmark of this study.

#### 3.4.2. NucleusSeg Dataset

The second dataset was introduced in [69] and then used in [67]. It is composed of 61 RGB images with a resolution variable of approximately 1000×700 cancer cells/nuclei taken from the Huh7 and HepG2 regions. Koyuncu et al. acquired images under a Zeiss Axioscope fluorescent microscope with a Carl Zeiss AxioCam MRm monochrome camera with a 20× Carl Zeiss objective lens. For the Hoechst 33258 fluorescent dye, a bisbenzimide DNA intercalator can be observed in the blue region upon UV region excitation. Hoechst 33258 dye was excited at 365 nm and the blue light (420 nm) emitted was acquired [69]. Therefore, the bright color in this dataset was not white but blue. Therefore, this dataset was preprocessed to extract the blue channel of the image, to obtain a grayscale image representative of fluorescence. This dataset was not used to train the model, because it did not include a variety of neuroblastoma datasets. However, it was used as a test set to examine the generalization capacity of the algorithms.

#### 3.4.3. ISBI 2009 Dataset

The third dataset was introduced in [70] and is composed of 46 grayscale images with a resolution variable of about 1350×1000 U2OS cells, created explicitly as computer vision benchmarks. To the best of our knowledge, the authors did not declare the microscope used and the IF methods involved. Moreover, the dataset was segmented by hand by the authors, making the handmade segmentation in GIMP files publicly available. Then, the dataset was preprocessed to transform the data images and the author’s segmentation into explicit images.

## 4. Proposed Method

### 4.1. Why a New Approach Is Needed

The previous algorithms were all based on artificial neural networks, and they performed well under optimal conditions. However, the literature shows that deep neural networks (DNN) can be affected by adversarial attacks [71]. An adversarial attack is a small perturbation (often called adversarial noise) introduced and tuned by a machine-learning algorithm to induce misclassification in the network. Segmentation neural networks are not immune to these types of attack. For example, in [72], it was proven that an adversative attack can fool ICNet [73], which provides semantic segmentation by controlling an autonomously driving car. The changes in the adversative algorithm applied to the input image are so subtle that a real-world light imperfection or a camera sensor not working properly can recreate the adversative pattern, leading to an accident. This unlucky (but possible) scenario reveals the second major problem of these algorithms: they are black boxes, and in many cases, after an accident, the best solution is to train the network again, hoping that no similar issues will affect the network in the future. Therefore, the concept of explainable artificial intelligence (XAI) is becoming increasingly popular. In brief, XAI is an algorithm designed to determine its actions and eventually correct them. This is of crucial importance for the real-world application of deep learning in fields such as robotics, automation, and medicine, because an XAI can be fixed after an error, and a human can eventually be held accountable for its errors. Interpreting results in medical imaging topics is a crucial task for an accurate medical report. An exhaustive survey of recent XAI techniques in medical imaging applications was presented by Chaddad et al. in [74]. They presented several popular medical image XAI methods, regarding their performance and principles. Therefore, they emphasized the contribution of XAI to problems in the medical field and elaborated on approaches based on XAI models for better interpreting such data. Furthermore, this survey provides future perspectives to motivate researchers to adopt new XAI investigation strategies for medical imaging.

These assertions include the formulation of new methods, in which the DNN performance is guaranteed, and the correct approach of XAI must be considered. Therefore, our strategy defines a new method based on neuronal electric activity in brain, and which has formal correctness and good DNN performance, as well as in the presence of adversative noise.

### 4.2. Wired Behaviors in Neuronal Electric Activity

The crucial role of neuronal electric activity in brain cognitive processes is well known, for this reason it is important to describe neuronal networks in terms of wired behavior. Then, the first step is to determine a neuron model able to simulate neuronal electric activity. Use of spiking neuronal networks in signal processing is a new frontier in pattern classification, where efficient biologically inspired algorithms are used. In particular, Lobov et al. in [75] showed that, in generating spikes, the input layer of sensory neurons can encode the input signal based both on the spiking frequency and on the latency. In this case, it implements learning working correctly for both types of coding. Based on this assumption, they investigated how a single neuron [76] can be trained on patterns and then build a universal support that can be trained using mixed coding. Furthermore, they implemented a supervised learning method based on synchronized stimulation of a classifier neuron, with the discrimination task among three electromyographical patterns. Thier methodology showed a median accuracy 99.5%, close to the result of a multi-layer perceptron trained with a backpropagation strategy.

There are many neuronal models, but the most accurate is the Hodgkin–Huxley model [77]. However this model is made up of several ODEs (ordinary differential equations) and some PDEs (partial differential equations). Owing to its complexity, less accurate but simpler models are preferred to the Hodgkin–Huxley model for many applications. A popular model in the application is the *Leaky, Integrate and Fire model* [78] (hereafter LIF). Moreover, an interesting methodology was proposed by Squadrani et al. in [79], the authors planned a hybrid model, in which current deep learning methodologies are merged with classical approaches. The Bienenstock–Cooper–Munro (BCM) model created for neuroscientific simulations is finding applications in the field of data analysis, recording good performances.

The main equation of the LIF model is

$$I\left(t\right)=I_{R}+I_{C}$$

where I(t) is the external current, $I_{R}$ is the resistor current, and $I_{C}$ is the capacitor current. Then, it can be observed that defining *V* as the membrane potential, then $I_{C}=C\frac{dV}{dt}$ and $I_{R}=\frac{\Delta V}{R}=\frac{V-V_{rest}}{R}$. Then, it holds

$$I\left(t\right)=\frac{V-V_{rest}}{R}+C\frac{dV}{dt}$$

and then

$$C\frac{dV}{dt}=-\frac{V-V_{rest}}{R}+I\left(t\right)$$

multiplying by *R*

$$RC\frac{dV}{dt}=\tau \frac{dV}{dt}=-\left(V-V_{rest}\right)+RI\left(t\right)$$

where τ=RC is the membrane time constant. Observing that $\frac{dV}{dt}=\frac{d(V-V_{rest})}{dt}=\frac{d\;\Delta V}{dt}$, often the previous equation takes the form

$$\tau \frac{d\;\Delta V}{dt}=-\Delta V+RI\left(t\right)$$

for this reason it can be assumed $V_{rest}=0$ (even if experimental findings suggest that is not the case).

However, this differential equation alone cannot model neuronal activity, because it is observed that if the neurons reach a threshold potential, they “fire”, which means that they come back to the resting potential $V_{rest}$. Moreover, they keep this value for an interval of time called the refractory time. For this reason, we add to the ODE model the condition that if *V* reaches the value $V_{thr}$, then *V* is reset to $V_{rest}$ and keeps this value for the refractory time $\tau_{r}$. It may seem that neuron connectivity has no role in the network activity, but this is not the case, because the term I(t) is still unknown. This term usually includes the synaptic connections. For example, in [80], given a neuron *i*, this term takes the form

$$I_{i}\left(t\right)=\tau \sum_{j=1}^{N}J_{ji}\sum_{k}\delta (t-t_{j}^{k}+D)$$

where $J_{ji}$ is not zero if there is a connection starting from the neuron *j* and arriving at neuron *i*, and in particular $J_{ji}>0$ if the connection is excitatory and $J_{ji}<0$ if the connection is inhibitory. The term $\delta (t-t_{j}^{k}+D)$ says that if the neuron *j* fires at the time $t_{j}^{k}$, then it generates a reaction that is a delta distribution. In conclusion, supposing *G* is the connectome of the network, $J_{hj}=0$ if and only if ${\left(A_{G}\right)}_{ji}=0$. Therefore, the key role of connectivity in network dynamics is clear.

### 4.3. Segmentation

Segmentation of an image is the task of dividing the area of an image into subregions. A classic example of segmentation is when an algorithm is asked to mark an image area occupied by a given object with color. In this case, the objects are cells/nuclei, two main approaches can be used for segmentation:

1. The binary approach returns a binary image, in which every pixel is white if it belongs to an object and black otherwise. This approach considers all cells and nuclei as a single object.
2. The object-by-object approach assigns a different color to each object. Consequently, cells/nuclei are distinguishable and, in general, are numbered with natural numbers.

For a long time, this operation has been performed manually, and the datasets contain this information. We used the publicly available dataset introduced in Ref. [65]. This dataset contains 79 annotated nuclei from nuclei images of tissues stained with fluorescent antibodies. In particular, Ref. [65] subdivided the dataset into 41 images for the training set and 38 images for the test set.

In general, the images in this dataset do not have exactly the same resolution (however, the resolution of each image is approximately 1200 × 1000). Therefore, we assumed that each image had been properly rescaled before processing.

### 4.4. The Proposed Model

We propose an explainable artificial intelligence method based on mask transformation. Its main core is a module in which a set of neurophysiologic-based agents generate results. The algorithm comprises six main parts, as shown in Figure 2:

1. *Preprocessing phase*: prepares the images for the next steps.
2. *Watershed analysis*: splits cells/nuclei using the well-known watershed transformation [65].
3. *Two steps of splitting and merging phase*: improves watershed separation;
4. *Pattern extraction phase*: from the cells/nuclei masks, the contour was extracted to run the neuronal model.
5. *Neuronal model phase*: manipulate the contour of the mask using neuronal agents;
6. *Postprocessing phase*: Some thresholding algorithms improve the mask precision.

### 4.5. Model Description

The main goal of the proposed algorithm is to segment bright (colored) cells/nuclei on a dark background in grayscale images. However, the dataset detailed in the previous sections consists of blue or green RGB images. The underlying concept is that the dataset is processed to extract the brightness of cells/nuclei, to obtain a grayscale image suitable for further processing. For example, Hoechst staining data were extracted from the dataset NucleusSegData [67], which was obtained using U.V. light, and the color of such images was blue. Therefore, in this case, the blue channel was extracted from the image to obtain a grayscale image with the desired properties. Every step is linked to the others using a constant called the scale factor (since now sf), which is defined as

$$sf=\frac{(Height+Width)/2}{\mu_{ds}}$$

where height and width are the dimensions of the image in pixels and $\mu_{ds}$ is the average value of image resolution for the neuroblastoma dataset (ex $\mu_{ds}=1110$). This value is introduced to provide scale-invariant properties for the algorithm. In the subsequent paragraphs, it will be necessary to estimate a real number to the nearest odd or even number. Therefore, we introduce the following functions:

odd(x)=2⌈(x−1)/2⌉+1

and

even(x)=2⌈x/2⌉

where ⌈x⌉ denotes the ceiling approximation. These functions are often used to filter dimensions.

The first phase is the preprocessing (see Figure 2a), in which a rough contour is evaluated. Thus, the image is enhanced, and a first rough segmentation is performed. Specifically, the grayscale image is sharpened through a convolution process [54,81] with this kernel:

$$\left[\begin{array}{ccc} -0.5 & -0.5 & -0.5\\ -0.5 & 4.5 & -0.5\\ -0.5 & -0.5 & -0.5 \end{array}\right]$$

Then, a median blur is applied [54] with a 3 × 3 size. Defining as Img the result of the previous processes, a chain of Gaussian filters [54] is then applied, with a standard deviation 5×sf

$$\mu =gaussian\left(Img,5\times sf\right)$$

$$\mu_{2}=gaussian\left(\mu ,5\times sf\right)$$

Next, a type of low-pass filter is calculated using the formula

$$\sigma_{0}=\sqrt{\left|\mu_{2}-\mu^{2}\right|}$$

where $\mu^{2}$ is the pixel-wise multiplication of image μ with itself. The $\sigma_{0}$ is normalized to be encoded as an 8-bit depth image using the following formula:

$$\sigma =255\times \frac{\sigma_{0}}{\max\left(\sigma_{0}\right)}$$

Finally, Otsu segmentation [45] is performed on the image σ using OpenCV [60]. Using the above segmentation, a watershed module performs a transformation [59] with the OpenCV function [60] to detect single cells and nuclei (see Figure 2a). The results at this stage are poor, and further steps are required to achieve optimal performance. The main problems related to this first segmentation concern cells/nuclei that are over segmented (the mask is bigger than the ground truth) or poorly split (many cells/nuclei that should be identified as distinct are classified as a single object). Therefore, we introduced a two steps of splitting and merging task (see Figure 2b). This routine assumes that the size of cells/nuclei in the image is uniform, and if an object is significantly larger than the average, it is a cluster. Every time the cell/nuclei splitting routine identifies a cluster, it starts trying to split it into a number of cells/nuclei coherent with its surface in relation to the average object size. In detail, the algorithm starts from the grayscale image Img, for which is calculated $\sigma_{0}$, as proposed in Equations (1) and (2). Then, an intermediate image is calculated as

$$G=0.5\times Img+0.5\times 255\times \frac{\sigma_{0}}{\max\left(\sigma_{0}\right)}$$

Then, the segmentation calculated in the previous phase (with *N* cells/nuclei) is loaded, and (defining $A_{i}$ as the area of object *i*), we calculate $A_{avg}$ as the average area of an object segmented as

$$A_{avg}=\frac{\sum_{i=1}^{N}A_{i}}{N}$$

Then, for each object *i*, we calculate the ratio

$$r_{i}=\frac{A_{i}}{A_{avg}}$$

This ratio encapsulates the idea that if an object is two or three times larger than the average, then it is likely to be composed of two or three different cells/nuclei. If $r_{i}>1.5$, the algorithm attempts to split the object. In this case, the object mask is preprocessed three times through dilatation with size odd(5×sf) iterations. Then, an iterative process attempts to optimize the number of cells/nuclei using the following routine:

1. Given $V_{up}$ (initialized at 190) and $V_{down}$ (initialized at 0) is calculated $V_{tresh}=0.5\times V_{u}p+0.5\times V_{down}$
2. On the image *G* is performed a threshold at $V_{tresh}$, creating a binary mask
3. The threshold binary mask is processed three times with an size odd(5×sf) and two times with an size odd(5×sf)
4. $\rho_{i}$ is calculated as the number of sufficiently large connected components of thresholding mask
5. The distance is calculated $\delta_{i}=\left|r_{i}-\rho_{i}\right|$
6. If $\delta_{i}$ is minimal, this configuration is saved
7. In any case, if $r_{i}>\rho_{i}$, then $V_{up}=V_{tresh}$ else $V_{down}=V_{tresh}$
8. Return to Step 1, until the desired number of iterations is reached (in this contribution, this number is 10).

In conclusion, the optimal segmentation in terms of $\delta_{i}$ is taken as the result of the splitting procedure. However, the result of this splitting procedure is undersegmentation (any regions marked as the ground truth are not present in segmentation). To combine the positive aspects of the two segmentations, we implement a fusion task between the segmentation results of the watershed module and the split-and-merge task. This process is performed by taking the segmentation computed before the splitting procedure as input. Each marked pixel of the input segmentation is then assigned to the nearest object of the split segmentation. This approach makes it possible to obtain the number of cells/nuclei for splitting segmentation using the binary mask of the original segmentation. This splitting–merge cycle is iterated twice, to improve the segmentation. Next, the contour extraction phase starts, and the result is a collection of binary masks, one for each object. Each mask is dilated three times with a kernel of the size even(20×sf) and then eroded three times with a kernel of size odd(5×sf) (Pattern extraction phase in Figure 2c). The mask is then subdivided into 30 radial bins, and the mean radius is calculated for each bin. A collection of 30 points following the contour of the mask is obtained from the bin angle and the average radius. This phase is shown in Figure 2c as a contour–extraction module and then, the generated contour provides an idea of the displacement of an object, but this is not sufficiently precise to challenge the state-of-the-art models.

Therefore, each point of the contour is associated with a neuronal agent in the neuronal model phase (see Figure 2d), which moves along the line starting from the center of the object and passing through the original contour point. This agent is then a 1D agent that can move in the direction of the center or away from the center. The motion of the agent is controlled by the simple idea that the agent is repulsed by high grayscale values in the input image but is attracted by the segmentation mask previously computed. These conditions create a system that converges on the position of equilibrium, which is the actual contour of the object. The agent is the neuronal network introduced in Section 4.2 and built into NEST [82], with eight neurons distributed in three layers (see Figure 3). The first layer (L1) is the input layer and is composed of four neurons: the LS, LE, RE, and RS. To calculate the intensity of the current to which these neurons are subjected, we need to calculate the images

(1) $$gray=gaussian\left(EqHist\left(Img\right),30\times sf\right)$$

where EqHist is a function of histogram equalization.

In conclusion, for each pixel $p=(p_{x},p_{y})$, it is possible to calculate

$$E\left(p_{x},p_{y}\right)=3000\times \left(\frac{\sum_{i=p_{x}-sd}^{px+sd}\sum_{j=p_{y}-sd}^{py+sd}mask_{filt}\left(i,j\right)}{2\;sd^{2}}\right)$$

$$S\left(p_{x},p_{y}\right)=3000\times \frac{3}{8}\times \left(\frac{\sum_{i=p_{x}-sd}^{px+sd}\sum_{j=p_{y}-sd}^{py+sd}gray(i,j)}{sd^{2}\;gray_{avg}}\right)$$

where

$gray_{avg}$ is defined as the average grayscale value of gray. The defined mask as the binary mask obtained by the previous steps is dilated three times with a kernel of size even(10×sf).

(2) $$mask_{filt}=\frac{2\times gaussian(mask,15\times sf)+0.1}{1+0.1}$$

sd=even(10×sf), the 3000 value is the stimulation intensity, and 3/8 is an empirical value fixed for all experiments.

Now, if $p_{0}$ is the center of the object and $p_{c}$ is the contour position, for each agent, the coordinate λ is introduced, such that the position of the agent can be expressed as

$$p_{a}\left(\lambda \right)=\lambda p_{c}+\left(1-\lambda \right)p_{0}$$

We defined the stimulation current for the neurons LS ($S_{LS}$), RS ($S_{RS}$), LE ($E_{LE}$), and RE ($E_{RE}$) as

$$S_{LS}\left(\lambda \right)=S\left(p_{a}\left(\lambda -0.2\right)\right)$$

$$S_{RS}\left(\lambda \right)=S\left(p_{a}\left(\lambda +0.2\right)\right)$$

$$E_{LE}\left(\lambda \right)=E\left(p_{a}\left(\lambda -0.2\right)\right)$$

$$E_{RE}\left(\lambda \right)=E\left(p_{a}\left(\lambda +0.2\right)\right)$$

The neuron LS has an excitatory synapse on the neuron LI and the neuron RM; in contrast, the neuron RS has an excitatory synapse on the neuron RI and the neuron LM. The LE and RE neurons have excitatory synapses on the LM and RM neurons, respectively. The role of this first layer is to “perceive” the image configuration and send it to the next layers.

The second layer (L2) is made up of two inhibitory neurons, LI and RI, which have an inhibitory synapse on the neurons LM and RM. Their role is to inhibit the motor neurons LM and RM when the LS and RS neurons are activated. Neurons LM and RM, included in layer 3, are responsible for the motion of the agent. The speed of the agent is calculated as

$$speed=0.06\left(Spikes_{RM}-Spikes_{LM}\right)$$

where $Spikes_{RM}$ and $Spikes_{LM}$ are the number of spikes in the simulation window (5 ms). Following the previous steps, each agent reaches the convergence point. After this, the agent mask is postprocessed in the post-processing phase with a standard binary segmentation [62] inside the masks, which has a good performance, because its content is bimodal (see Figure 2e). However, some cells/nuclei are still clustered, and for this reason, a final splitting and merge cycle is performed. This final splitting differs from the previous splitting because it is based on the distance transform L2 [83].

### 4.6. Evaluation Criterion and Metrics

Hereafter, only the binary segmentations of the methods discussed will be evaluated, even if some of them return object-by-object segmentation. In general, a bit of binary object segmentation can be called positive if the segmentation shows that there is an object on it, or negative if not. To evaluate the accuracy of binary segmentation, there are many metrics with slightly different interpretations, but every metric proposed is based on four sets: true positives (TP), true negatives (TN), false positives (FP), and false negatives (FN). The TP set is the set of pixels positive for the ground truth and the prediction. Similarly, the TN set is the set of pixels negative for the ground truth and the prediction. The FP set is the set of pixels that are positive for the prediction and negative for the ground truth. Analogously, the FN set is the set of pixels negative for the prediction and positive for the ground truth. The metrics used are as follows:

- *Intersection over Union* (IoU): This is defined as $\frac{TP}{TP\;+\;FP\;+\;FN}$ and is one of the most balanced metrics.
- *F1-score* is defined as $\frac{2TP}{2TP\;+\;FP\;+\;FN}$ and can be proven to be almost proportional to the IoU.
- *Accuracy* is defined as $\frac{TP\;+\;TN}{TP\;+\;FP\;+\;FN\;+\;TN}$ and is one of the most popular metrics for machine learning. However, in object segmentation tasks, this metric can be biased in cases of sparse cells/nuclei; in these cases, the number of negative pixels can be much greater than the number of positive pixels. This means that even if the prediction is fully negative (every pixel is negative), if the ground truth ratio P/N tends to 0, then the accuracy tends to 1.
- *Sensitivity* is defined as $\frac{TP}{TP\;+\;FN}$ and can be biased if the ground truth ratio N/P tends to zero.
- *Specificity* is defined as $\frac{TN}{TN\;+\;FP}$ and can be biased if the ground truth ratio P/N tends to zero.

## 5. Results

We tested NeuronalAlg against three datasets, with and without adversative noise and a ground truth as a baseline, to evaluate the performance our method. Table A1, Table A2, Table A3, Table A4, Table A5, Table A6, Table A7, Table A8, Table A9, Table A10, Table A11, Table A12, Table A13, Table A14 and Table A15 show that the method reported equivalent results to NNs and outperformed them in the presence of adversative noise (fast gradient sign method). Therefore, NeuronalAlg achieved a notable performance in cell/nuclei segmentation and, contrary to NN, it does not require a training phase to evaluate other datasets.

### 5.1. Adversative Noise

Using the fast gradient sign method (FGSM) methodology in [73], we performed an adversative attack on the network U-Net. The FGSM epsilon value was set to 0, 0.01, 0.025, 0.05, 0.1, and 0.2. Figure 4, Figure 5 and Figure 6 show three samples of images related to the Neuroblastoma dataset with different PSNRs. U-Net, in the absence of noise, performed very well (Figure 4, second row). However, in the presence of a slight noise (Figure 5, second row), the U-Net segmentation started to exhibit large holes in the cells/nuclei, and the phenomena become worse when the noise became consistent (Figure 6, second row). In these cases, the segmentation almost disappeared and no object was shown. One of the most common criticisms regarding adversative noise is that the ML model that generates it is trained on the network and is strongly focused on the analyzed network. Thus, the images obtained by the FGSM trained on the U-NET were evaluated using the KG network and mask R-CNN network. The result was that the KG network was very resistant to intermediate noise (Figure 5, third row), but often returned no segmentation with high noise values (Figure 6, third row). The mask R-CNN showed a good performance under every noise condition, even if some cells/nuclei were lost with strong noise (Figure 6, third row). The best performance was achieved using the proposed model. Indeed, the segmentation was consistent for low and intermediate noise (Figure 4 and Figure 5, third row), and it only exhibited a few holes if the noise was strong (Figure 6, third row).

### 5.2. Results for the Neuroblastoma Dataset

Judging algorithms based on a few images is not a good practice, so we conducted a quantitative investigation of the results of the algorithms with adversative noise with peak signal-to-noise ratio (PSNR) values of 100.0, 40.1, 32.7, 26.9, 21.1, and 15.7. The algorithms were evaluated using a test set of 38 images. Each image was compared with the ground truth, to compute the metrics of intersection for the union, F1-score, accuracy, sensitivity, and specificity. The results are plotted in Figure 7 and summarized in Table A1, Table A2, Table A3, Table A4 and Table A5 (in the Appendix A.2). It can be observed from Figure 7a and Table A1 that, in terms of IoU for PSNR 100.0 (no noise), the U-Net had the best performance, with an IoU of 0.718, followed by the KG network with an IoU of 0.712, the proposed Neuronal algorithm with an IoU of 0.708, and finally the Mask R-CNN with an IoU of 0.682. However, by adding adversative noise, the situation changed and U-Net showed the steepest descent, but these results can be explained by the fact that adversative noise was created ad hoc for this network. The KG network seemed to maintain a good performance with a low amount of adversarial noise, but with a PSNR less than 25, the IoU dropped to less than 0.32. The other algorithms outperformed the mask R-CNN for low noise, but when adding a strong noise, the IoU had a slower decay than the other DNNs, even when the maximum adversarial noise reached an IoU of 0.21. The proposed model outperformed the DNN in terms of noise resistance, because with a PNSR = 15.7 (the highest noise evaluated), it achieved the lowest IoU value of 0.55, which was more than twice that of the other algorithms.

The F1-score (Figure 7b and Table A2) had a behavior equivalent to that of the IoU, and all the considerations for the IoU remained for the F1-score. Accuracy (Figure 7c and Table A3) and all other metrics must be carefully analyzed. If U-Net, KG network, and mask R-CNN produce false classifications, they are more prone to false negatives (FN). In contrast, the proposed neural algorithm is more prone to producing a false positive (FP) in the case of false classification.

This translates into the observation that DNNs usually have more background (negatives) than the ground truth, and they are undersegmented because the cells/nuclei parts are cut by the algorithm, being classified as background. On the other hand, the proposed model showed oversegmentation, because part of the background was classified as cells/nuclei. This simple observation slightly conditioned the accuracy, because in the undersegmenting algorithms, the number of TNs is more significant than in an oversegmenting algorithm. However, the overall graph confirms that the proposed model was more resistant to adversarial noise.

The previous event became clearer in terms of sensitivity (Figure 7d and Table A4) and specificity (Figure 7e and Table A5). Indeed, the proposed neuronal algorithm outperformed the DNN algorithms in terms of sensitivity (because in this case, the FN was very small), whereas the DNN algorithms outperformed the proposed algorithm in terms of specificity (because in these cases, the FP was very small). However, the specificity case was very curious, because it seemed that the greater the noise, the better the specificity. The answer is found in Figure 4, Figure 5 and Figure 6, in which many DNN predictions with strong noise are almost positive-free (black segmentation); in this case, TP = 0 or FP = 0, which means that it has a specificity = 1, regardless of the value of TN. In these cases, with greater noise, positive-free segmentation is more likely to be achieved with a higher specificity. For these reasons, more balanced metrics (such as IoU and F1-score) are preferred for the interpretation of results.

Neural networks such as the mask RCNN [52] are endowed with a data augmentation module [84] that improves the performance for high noise values. A series of tests were conducted on the networks U-Net [48] and KG Network [51]. The results of these tests can be seen in Figure 7, plotted with dashed lines. For the U-Net (Figure 7, orange dashed line) we obtained a significant improvement in terms of performance, reaching the performance of the KG Network. This can be explained by the fact that such a network was used to compute the adversarial perturbation. However, significant improvements were not observed for the KG Network (Figure 7, green dashed line). However, even with data augmentation, the proposed method still outperformed the reference neural networks in the case of high noise.

Moreover, for a more appropriate evaluation of the proposed method for the Neuroblastoma dataset, a selection of results is reported in Appendix A.1 Figure A1, Figure A2 and Figure A3. They depict the average, best, and worst case for the adopted methods with three PSNRs (100.0, 32.7, and 15.7).

### 5.3. Results on the Nucleussegdata Dataset

Generally, the principal critique that motivates moving towards non-deep-learning approaches is that they must be tuned on a single dataset (in the worst cases, on a single image) to achieve performances comparable to neural networks. Therefore, further tests were performed. These tests attempted to quantify the generalization capability of the previously exposed algorithms. For example, the DNNs (U-Net, KG network, and R-CNN) segmented the images of the dataset NucleusSegData [67] without fine-tuning. Such an effect could be a limitation; however, it is a typical process in real-life applications. Indeed, in some cases, the DNN algorithm is connected to a camera that directly streams the image to the algorithm [72], and then the algorithm is applied to images that could differ substantially from the training and test sets used by the authors. In other cases, fine-tuning has to follow strict rules [85], which would make it impractical. Similarly, the neuronal model was used on the same dataset (NucleusSegData) without tuning any parameters. Table A6 presents the performance of the four models with the NucleusSegData dataset. Adversative noise was added to this dataset, with a PSNR of 100.0, 43.0, 34.5, 28.7, 22.6, and 16.6. The results are presented in Table A6, Table A7, Table A8, Table A9 and Table A10 (in the Appendix A.2) and Figure 8. Figure 8a,b with Table A6 and Table A7 show how the proposed model outperformed the NNs with high noise values. Figure 8c and Table A8 shows that the proposed model had a high accuracy for PSNR values greater than 16.6. However, for a PSC = PSNR equal to 16.6, the accuracy value decreased suddenly. This phenomenon was caused by the fact that the proposed model oversegmented with high values of noise; instead, the other networks were more prone to undersegmentation. The same phenomenon was the cause of the values of sensitivity and specificity shown in Figure 8d,e and Table A9 and Table A10, respectively. It can be seen (as for the previous dataset) that the proposed model had, in general, a higher sensitivity and specificity than the neural networks, with the feature that specificity increased when the noise PSNR increased. In general, these simulations showed better values than the Neuroblastoma case, which can be attributed to the similarity of the Neuroblastoma dataset to real-world data [65]. By contrast, NucleusSegData [67] is a less complex dataset. Finally, the proposed model showed a generalization capability comparable to that of the current state-of-the-art deep learning algorithms, countering the assumption that the explainable method implies manual parameter tuning, because this paper introduced an algorithm that had equivalent performances on all the datasets, without any parameter changes. This opens the way to explainable methods that would perform well on data qualitatively close to the datasets used in this paper, without any parameter changes required from the end user.

Moreover, for a more appropriate evaluation of the proposed method for the Nucleussegdata Dataset, a selection of the results is reported in Appendix A.1 Figure A4, Figure A5 and Figure A6. They depict the average, best, and worst cases of the adopted methods with three PSNRs (100.0, 34.5, and 16.6).

### 5.4. Results on the ISBI 2009 Dataset

This last evaluation was introduced to avoid the uncertainty regarding the accuracy of the method. As in the previous evaluation, adversative noise was adopted with the dataset with the following PSNR values: 100.0, 43.0, 34.5, 28.7, 22.6, and 16.6. The same trend was observed in the analysis of the ISBI dataset [70], as depicted in Table A11, Table A12, Table A13, Table A14 and Table A15 (in the Appendix A.2) and Figure 9a–e. The results show how the proposed model outperformed the NNs for high noise values and it had a high accuracy and sensitivity with PSNR values greater than 16.6. Moreover, it showed a phenomenon were the proposed model oversegmented for high values of noise; instead, the other networks were more prone to undersegmentation.

Moreover, for a more appropriate evaluation of the proposed method for the ISBI 2009 Dataset, a selection of results are reported in Appendix A.1 Figure A7, Figure A8 and Figure A9. They depict the average, best, and worst case for the adopted methods with three PSNRs (100.0, 33.3, and 16.6).

Although our method has formal correctness and a high performance, it requires computational improvements because it has a computation time approximately 10 times that of the DNN algorithms. However, in defense of this method, it must be noted that the neural networks run on hardware optimized for this task (GPUs). It has been shown that the speed increase of using a CPU instead of a GPU can reach a factor of 100 [86]. However, our neuronal agents are fully parallel and run on a CPU, which opens the way for GPU implementation. To date, the most promising approach has been neuromorphic computing [87]. This approach could reduce the number of iterations required to compute the neuron model using a hardware circuit that reproduces neuron behavior, drastically reducing the time required. Such hardware has already been successfully tested for a multitude of tasks [88]. For these reasons, computation times have been inserted to make the paper as complete as possible, but they are not representative of the performances that will be achieved on more targeted hardware in the following years.

## 6. Conclusions

From an investigation of new methods in image analysis, it can be noted that there has been a explosion of techniques based on machine learning and its high level of flexibility. However, comprehension of these results is usually obscure. Researchers have taken significant steps toward the direction of an explainable deep neural network. Frameworks such as SHAP [89] and LIME [90] can explain the most significant input variables that led a DNN to a classification. However, they do not solve particular problems linked to certain specific inputs. For example, if a NN is affected by adversarial noise, methods such as those previously described would tell us which pixels led the model to this decision. However, this gives us no insight into how to fix the problem itself. The only thing that can be done is to train the NN again and hope that the problem will not occur with another problematic input. This evidence suggests that methodologies based on machine learning, deep learning, and general methods oriented toward artificial intelligence are not a panacea for any problem present in the computational sciences.Nonetheless, many researchers continue to direct their research towards models that, on the one hand, do not involve the classic models of machine learning and, on the other hand, meet the performance of ML and have results that are more comprehensive. Hence, some efforts have been made to formulate formal methods that are detached from any reference to artificial intelligence and are comparable to the best performing and current artificial intelligence techniques. The planned contribution (NeuronalAlg) highlights a computational model based on deterministic foundations and not on a neuronal network. Our primary purpose was to demonstrate its formal correctness, easy exploration, and comparable results to the best-performing AI models in the presence of adversative noise. We demonstrated good qualitative results, and a more accurate analysis was carried out with a set of quantitative indicators (see IoU, F1-Score, accuracy, specificity, and sensitivity indexes in Table A1, Table A2, Table A3, Table A4, Table A5, Table A6, Table A7, Table A8, Table A9 and Table A10), which was necessary to compare them with the most common models of neuronal network. The analysis performed on the original images and modified with different noise models demonstrated reliable and robust results. The graphs and tables extracted from the experiments indicate inessential nature of the use of AI techniques, even when the application context requires formally correct models with demonstrable results. The indicators in the graphs and the data in the tables indicate an equivalence between the methods based on AI and methodologies containing neurophysiological models. Moreover, the explainability of the model comes from the fact that most parameters are semantically understandable. It is easy for a human to understand what is happening during the execution of the method. Therefore, we propose a model that appears to be a valid alternative to NN methods in contexts where reliability and robustness must be formally verifiable, even for negligible percentages of error, which is necessary to understand the reasons for their occurrence.

## Figures and Tables

**Figure 1:**
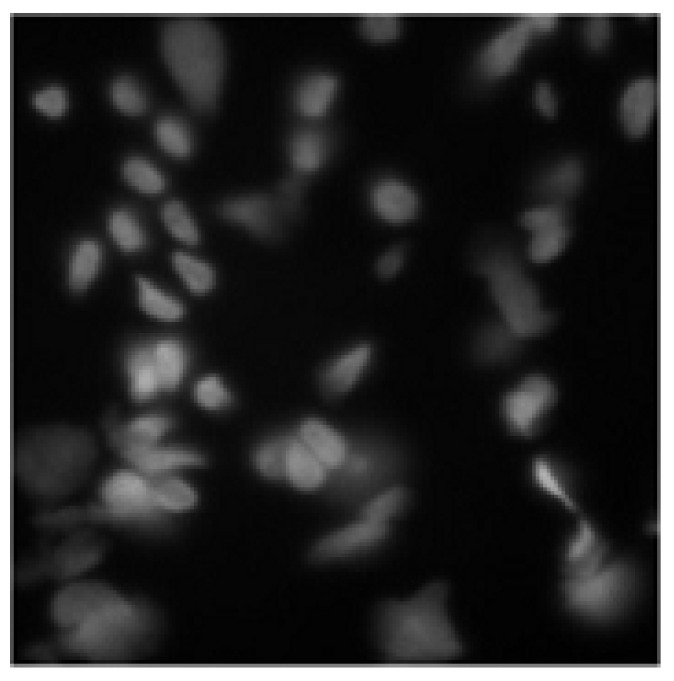
Example of a complex image in which some nuclei cannot be clearly distinguished from the cytoplasm.

**Figure 2:**
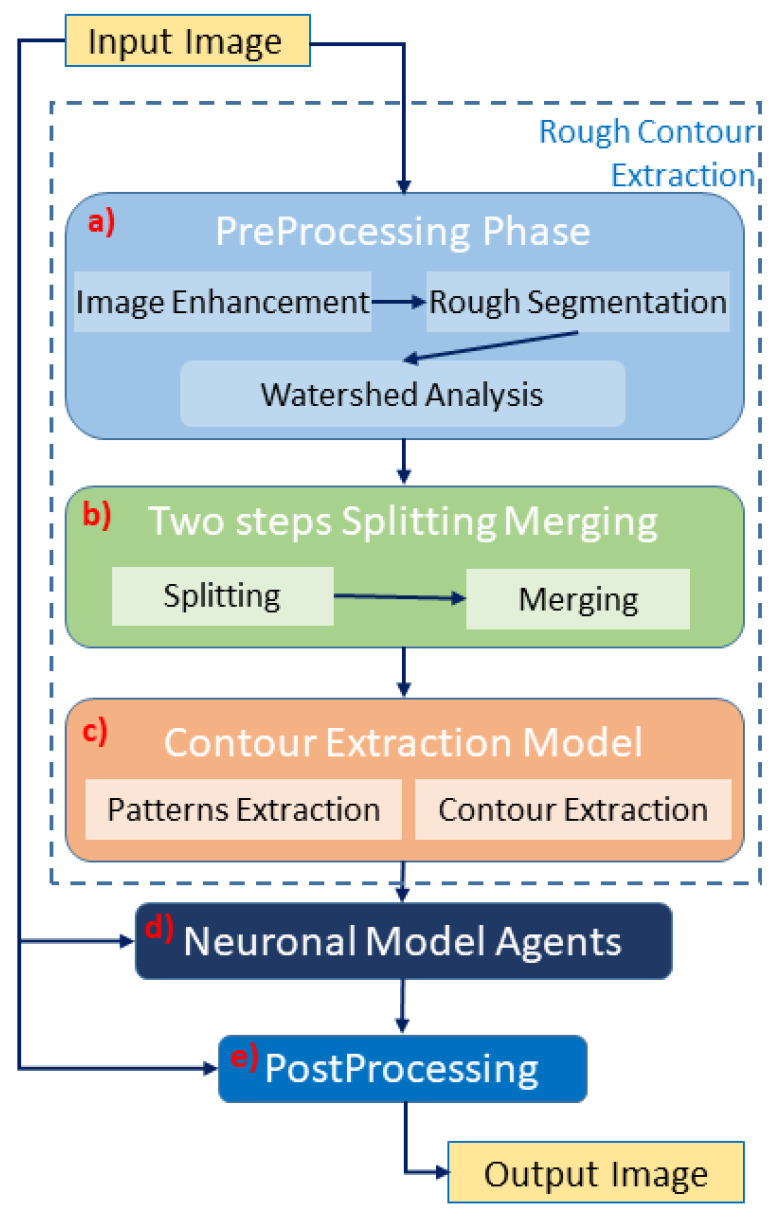
Sketch of our deterministic method (NeuronalAlg): (**a**) preprocessing module extracts a rough segmentation of the input image; (**b**) split and merge module as a first step for improving the previous segmentation; (**c**) last step of the rough segmentation; (**d**) main core of the task: neuronal method. This module improves the segmentation with a neuronal agent; (**e**) postprocessing phase, to extract the binary mask.

**Figure 3:**
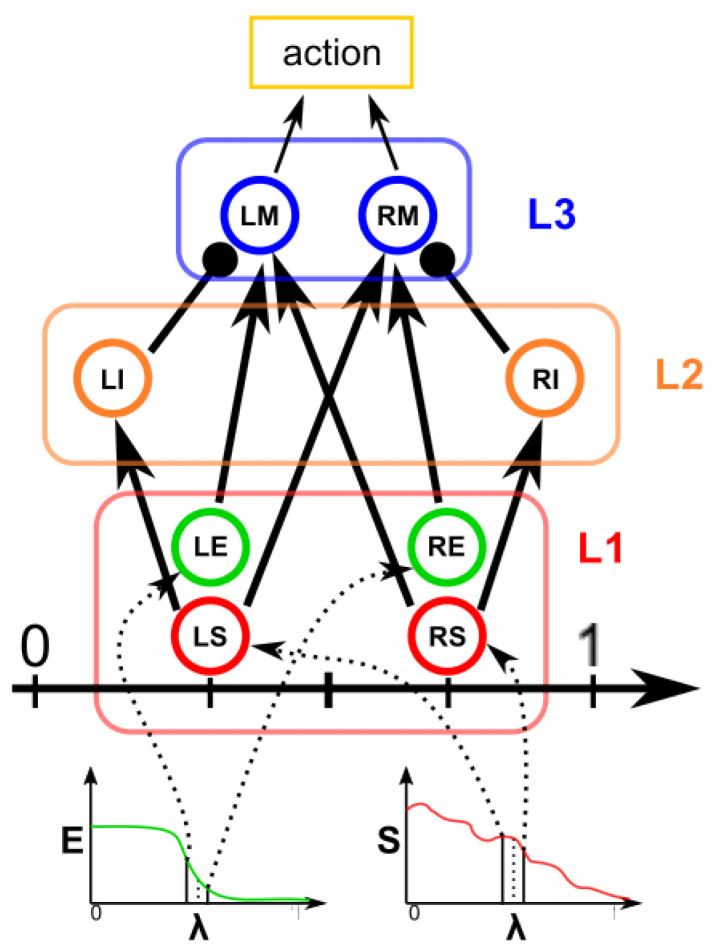
Sketch of the neuronal agent.

**Figure 4:**
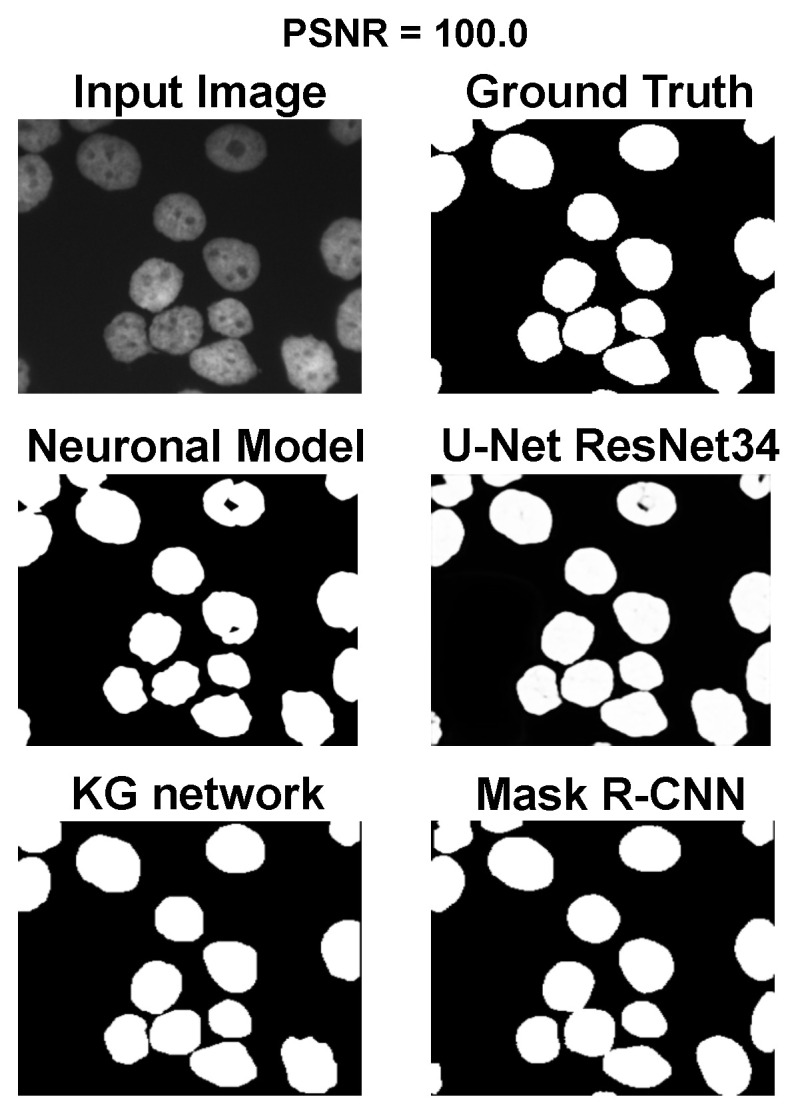
Results for an input image without noise.

**Figure 5:**
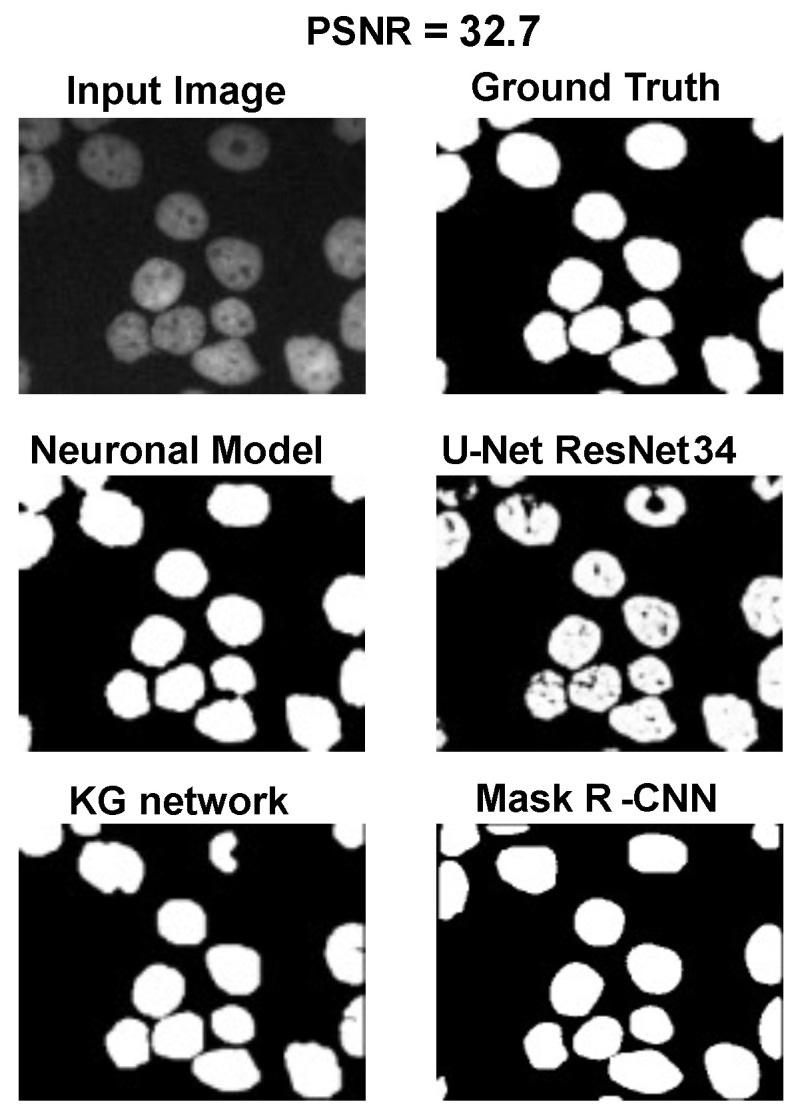
Results for an input image with a 32.7 PSNR.

**Figure 6:**
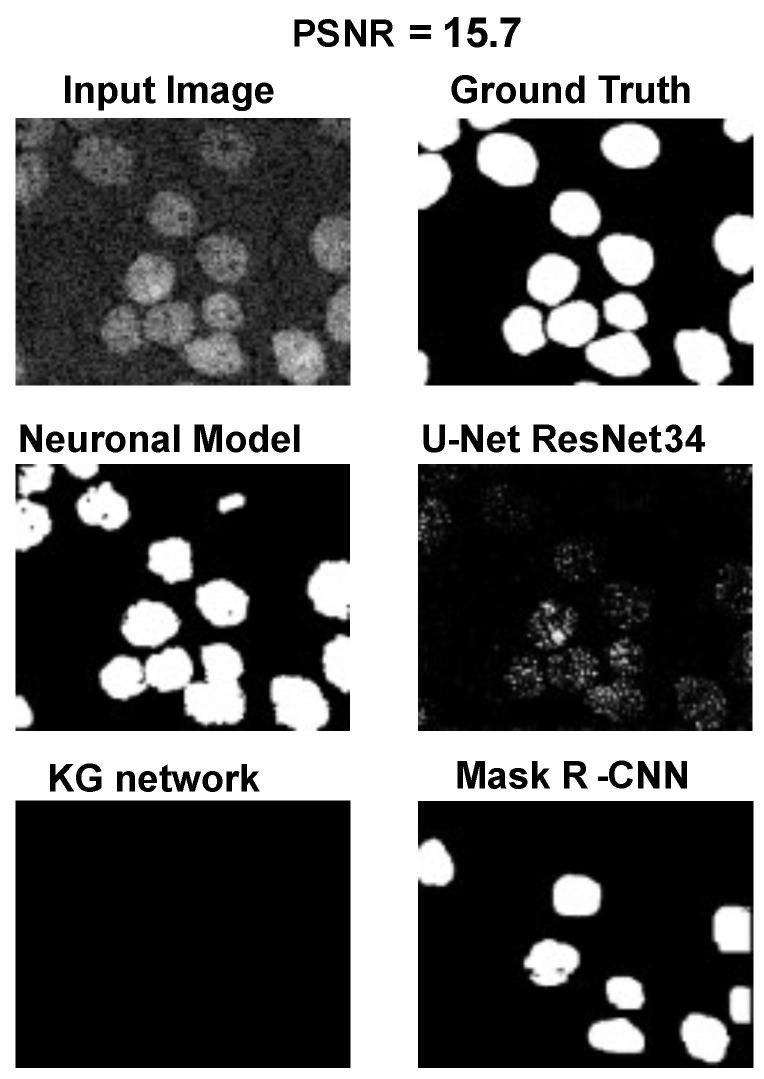
Results for an input image with a 15.7 PSNR.

**Figure 7:**
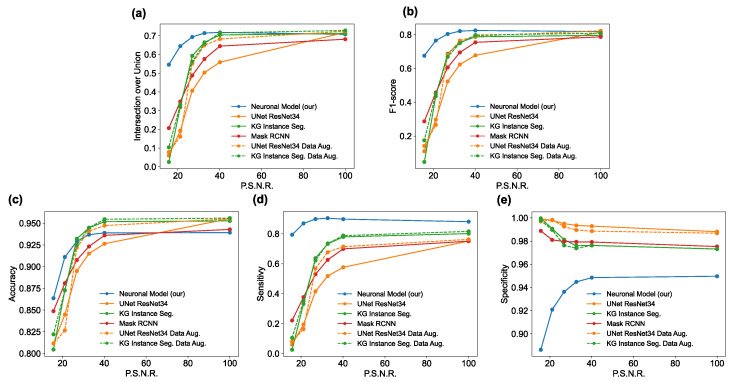
Performance in terms of IoU (**a**), F1-score (**b**), accuracy (**c**), sensitivity (**d**) and specificity (**e**) for each algorithm on the Neuroblastoma dataset.

**Figure 8:**
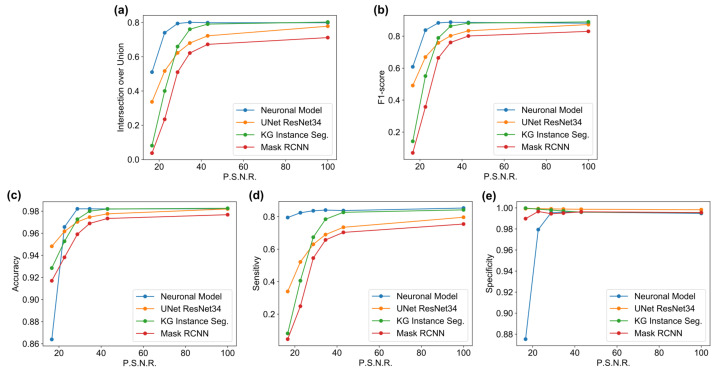
Performances in terms of IoU (**a**), F1-score (**b**), Accuracy (**c**), Sensitivity (**d**) and Specificity (**e**) for each algorithm on NucleusSegData dataset.

**Figure 9:**
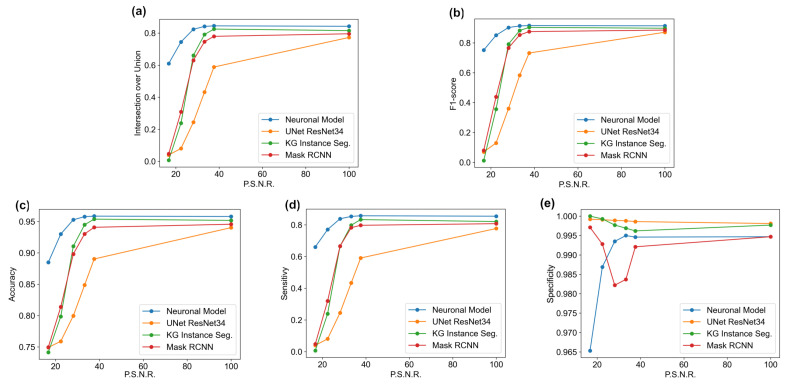
Performances in terms of IoU (**a**), F1-score (**b**), accuracy (**c**), sensitivity (**d**), and specificity (**e**) for each algorithm on the ISBI 2009 dataset [70].

**Figure A1:**
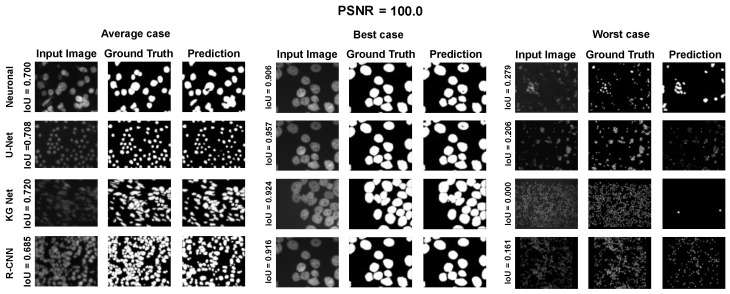
Average, best, and worst case for the Neuroblastoma Dataset with PSNR = 100.0.

**Figure A2:**
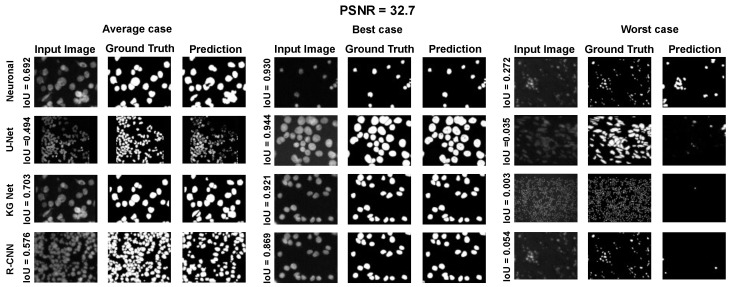
Average, best and worst case for the Neuroblastoma Dataset with PSNR = 32.7.

**Figure A3:**
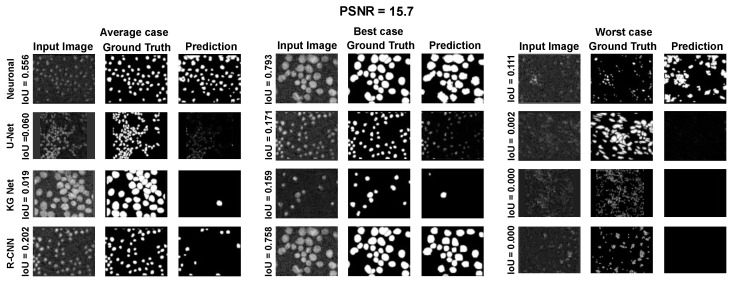
Average, best, and worst case for the Neuroblastoma Dataset with PSNR = 15.7.

**Figure A4:**
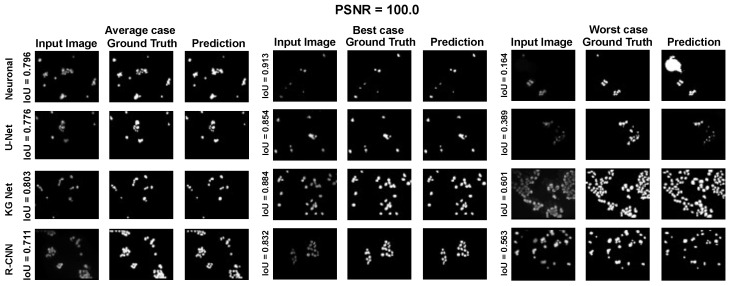
Average, best, and worst case for the dataset NucleusSegData with PSNR = 100.0.

**Figure A5:**
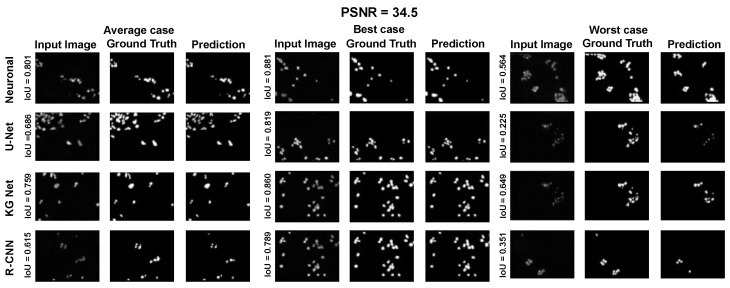
Average, best, and worst case for the dataset NucleusSegData with PSNR = 34.5.

**Figure A6:**
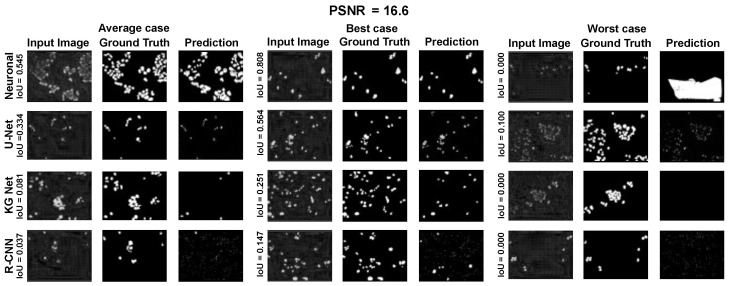
Average, best, and worst case for the dataset NucleusSegData with PSNR = 16.6.

**Figure A7:**
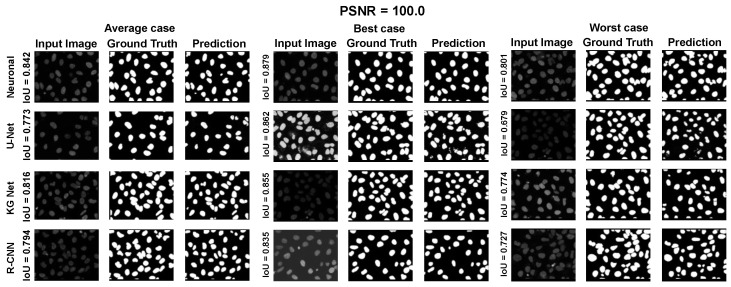
Average, best, and worst case for the dataset ISBI 2009 with PSNR = 100.0.

**Figure A8:**
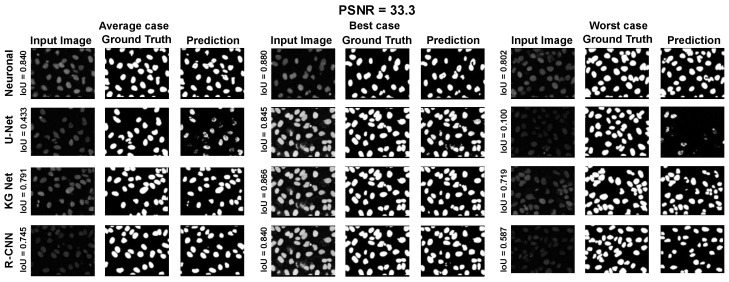
Average, best, and worst case for the dataset ISBI 2009 with PSNR = 33.3.

**Figure A9:**
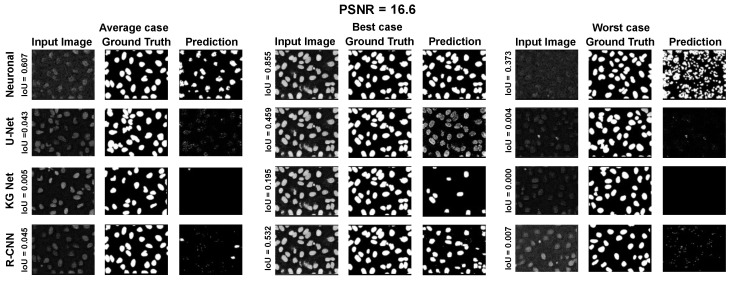
Average, best, and worst case for the dataset ISBI 2009 with PSNR = 16.6.

## Data Availability

NEUROBLASTOMA DATASET (accessed on 10 January 2022): An annotated fluorescence image dataset for training nuclear segmentation methods (https://www.ebi.ac.uk/biostudies/studies/S-BSST265); NUCLEUSSEGDATA DATASET (accessed on 10 January 2022): Object-oriented segmentation of cell nuclei in fluorescence microscopy images (http://www.cs.bilkent.edu.tr/~gunduz/downloads/NucleusSegData/); ISBI 2009 (accessed on 10 January 2022): Nuclei Segmentation In Microscope Cell Images: A Hand-Segmented Dataset And Comparison of Algorithms (https://murphylab.web.cmu.edu/data/).

## Appendix A

### Appendix A.2

**Table A1:** Intersection of Union values of the algorithms for different PSNR values of adversative noise (Neuroblastoma).

IoU	100.0	40.1	32.7	26.9	21.1	15.7
Neuronal Alg.	0.695	0.700	**0.695**	**0.673**	**0.614**	**0.549**
UNetRNet34	0.718	0.559	0.503	0.406	0.192	0.060
KG network	0.712	0.704	0.664	0.593	0.319	0.025
Mask R-CNN	0.682	0.645	0.576	0.489	0.348	0.207
UNet DAug.	0.7238	0.6829	0.6492	0.5518	0.1612	0.0784
KG DAug.	**0.7281**	**0.7159**	0.6584	0.5622	0.3323	0.1038

**Table A2:** F1-score values of the algorithms for different PSNR values of adversative noise (Neuroblastoma).

F1-score	100.0	40.1	32.7	26.9	21.1	15.7
Neuronal Alg.	0.805	**0.808**	**0.802**	**0.785**	**0.739**	**0.683**
UNetRNet34	0.815	0.678	0.623	0.523	0.297	0.111
KG network	0.796	0.787	0.754	0.688	0.435	0.047
Mask R-CNN	0.787	0.755	0.695	0.606	0.457	0.287
UNet DAug.	**0.8229**	0.7918	0.7648	0.6812	0.2662	0.1421
KG DAug.	0.8092	0.7976	0.7497	0.6688	0.4491	0.1751

**Table A3:** Accuracy values of the algorithms for different PSNR values of adversative noise (Neuroblastoma).

Accuracy	100.0	40.1	32.7	26.9	21.1	15.7
Neuronal Alg.	0.938	0.938	0.936	0.929	**0.910**	**0.879**
UNetRNet34	0.955	0.926	0.915	0.895	0.845	0.811
KG network	0.953	0.952	**0.945**	**0.932**	0.873	0.804
Mask R-CNN	0.943	0.936	0.923	0.908	0.881	0.849
UNet DAug.	0.9552	0.9473	0.941	0.9221	0.8266	0.8117
KG DAug.	**0.9559**	**0.9546**	0.9449	0.9249	0.8729	0.8221

**Table A4:** Sensitivity values of the algorithms for different PSNR values of adversative noise (Neuroblastoma).

Sensitivity	100.0	40.1	32.7	26.9	21.1	15.7
Neuronal Alg.	**0.848**	**0.855**	**0.853**	**0.849**	**0.816**	**0.778**
UNetRNet34	0.751	0.576	0.518	0.416	0.194	0.061
KG network	0.801	0.779	0.732	0.637	0.331	0.026
Mask R-CNN	0.751	0.699	0.626	0.530	0.377	0.221
UNet DAug.	0.7625	0.7142	0.6769	0.5685	0.1633	0.0805
KG DAug.	0.8161	0.7877	0.7354	0.6223	0.3512	0.1053

**Table A5:** Specificity values of the algorithms for different PSNR values of adversative noise (Neuroblastoma).

Specificity	100.0	40.1	32.7	26.9	21.1	15.7
Neuronal Alg.	0.953	0.954	0.952	0.942	0.926	0.903
UNetRNet34	**0.988**	**0.993**	**0.993**	**0.995**	**0.998**	0.999
KG network	0.973	0.976	0.976	0.981	0.991	**0.999**
Mask R-CNN	0.975	0.979	0.979	0.980	0.981	0.989
UNet DAug.	0.9868	0.9886	0.9895	0.9925	0.9983	0.9969
KG DAug.	0.9732	0.9762	0.9738	0.9763	0.9897	0.9977

**Table A6:** Intersection of Union values of the algorithms for different PSNR values of adversative noise (NucleusSegData).

IoU	100.0	43.0	34.5	28.7	22.6	16.6
Neuronal Alg.	0.798	**0.799**	**0.801**	**0.794**	**0.740**	**0.510**
UNetRNet34	0.778	0.722	0.680	0.622	0.516	0.337
KG network	**0.802**	0.790	0.760	0.659	0.400	0.081
Mask R-CNN	0.712	0.672	0.622	0.510	0.235	0.037

**Table A7:** F1-score values of the algorithms for different PSNR values of adversative noise (NucleusSegData).

F1-score	100.0	43.0	34.5	28.7	22.6	16.6
Neuronal Alg.	0.881	**0.886**	**0.888**	**0.883**	**0.838**	**0.608**
UNetRNet34	0.873	0.834	0.802	0.758	0.669	0.491
KG network	**0.889**	0.882	0.863	0.789	0.550	0.142
Mask R-CNN	0.830	0.801	0.761	0.664	0.357	0.070

**Table A8:** Accuracy values of the algorithms for different PSNR values of adversative noise (NucleusSegData).

Accuracy	100.0	43.0	34.5	28.7	22.6	16.6
Neuronal Alg.	0.982	**0.982**	**0.982**	**0.982**	**0.966**	0.864
UNetRNet34	0.982	0.978	0.975	0.970	0.962	**0.948**
KG network	**0.983**	**0.982**	0.980	0.973	0.953	0.928
Mask R-CNN	0.977	0.973	0.969	0.959	0.938	0.917

**Table A9:** Sensitivity values of the algorithms for different PSNR values of adversative noise (NucleusSegData).

Sensitivity	100.0	43.0	34.5	28.7	22.6	16.6
Neuronal Alg.	**0.853**	**0.838**	**0.841**	**0.836**	**0.824**	**0.795**
UNetRNet34	0.796	0.735	0.690	0.630	0.521	0.340
KG network	0.842	0.827	0.784	0.637	0.406	0.081
Mask R-CNN	0.755	0.703	0.657	0.545	0.249	0.046

**Table A10:** Specificity values of the algorithms for different PSNR values of adversative noise (NucleusSegData).

Specificity	100.0	43.0	34.5	28.7	22.6	16.6
Neuronal Alg.	0.995	0.996	0.996	0.996	0.979	0.875
UNetRNet34	**0.998**	**0.999**	**0.999**	**0.999**	**0.999**	0.999
KG network	0.996	0.996	0.997	0.998	**0.999**	**1.0**
Mask R-CNN	0.996	0.996	0.995	0.995	0.997	0.990

**Table A11:** Intersection of Union values of the algorithms for different PSNR values of adversative noise (ISBI 2009).

IoU	100.0	43.0	34.5	28.7	22.6	16.6
Neuronal Alg.	**0.842**	**0.845**	**0.841**	**0.823**	**0.744**	**0.610**
UNetRNet34	0.773	0.588	0.431	0.244	0.080	0.039
KG network	0.815	0.825	0.791	0.660	0.238	0.007
Mask R-CNN	0.795	0.780	0.746	0.630	0.308	0.047

**Table A12:** F1-score values of the algorithms for different PSNR values of adversative noise (ISBI 2009).

F1-score	100.0	43.0	34.5	28.7	22.6	16.6
Neuronal Alg.	**0.914**	**0.916**	**0.914**	**0.903**	**0.851**	**0.752**
UNetRNet34	0.871	0.732	0.583	0.360	0.130	0.070
KG network	0.898	0.904	0.883	0.792	0.356	0.012
Mask R-CNN	0.886	0.876	0.853	0.766	0.439	0.080

**Table A13:** Accuracy values of the algorithms for different PSNR values of adversative noise (ISBI 2009).

Accuracy	100.0	43.0	34.5	28.7	22.6	16.6
Neuronal Alg.	**0.958**	**0.959**	**0.958**	**0.953**	**0.930**	**0.885**
UNetRNet34	0.940	0.890	0.849	0.800	0.759	0.749
KG network	0.952	0.954	0.945	0.911	0.799	0.741
Mask R-CNN	0.946	0.941	0.930	0.898	0.814	0.750

**Table A14:** Sensitivity values of the algorithms for different PSNR values of adversative noise (ISBI 2009).

Sensitivity	100.0	43.0	34.5	28.7	22.6	16.6
Neuronal Alg.	**0.854**	**0.857**	**0.853**	**0.838**	**0.770**	**0.660**
UNetRNet34	0.777	0.591	0.433	0.245	0.081	0.040
KG network	0.821	0.833	0.798	0.665	0.239	0.007
Mask R-CNN	0.807	0.797	0.782	0.666	0.320	0.048

**Table A15:** Specificity values of the algorithms for different PSNR values of adversative noise (ISBI 2009).

Specificity	100.0	43.0	34.5	28.7	22.6	16.6
Neuronal Alg.	0.995	0.995	0.995	0.994	0.987	0.965
UNetRNet34	**0.998**	**0.999**	**0.999**	**0.999**	**0.999**	0.999
KG network	0.998	0.996	0.997	0.998	**0.999**	**1.0**
Mask R-CNN	0.995	0.992	0.984	0.982	0.993	0.997
